# PLG-007 and Its Active Component Galactomannan-α Competitively Inhibit Enzymes That Hydrolyze Glucose Polymers

**DOI:** 10.3390/ijms23147739

**Published:** 2022-07-13

**Authors:** Michelle C. Miller, Aurelio J. Dregni, David Platt, Kevin H. Mayo

**Affiliations:** 1 Department of Biochemistry, Molecular Biology & Biophysics, University of Minnesota Health Sciences Center, 6-155 Jackson Hall, 321 Church Street, Minneapolis, MN 55455, USA; mill0935@umn.edu (M.C.M.); dregn005@umn.edu (A.J.D.); 2 Bioxytran Inc., 75 2nd Ave., Suite 605, Needham, MA 02494, USA; david.platt@bioxytraninc.com

**Keywords:** amylase, amylopectin, enzyme, galactomannan, maltase, NMR, polysaccharide, protein, starch, sucrase

## Abstract

PLG-007 is a developmental therapeutic compound that has been clinically shown to reduce the magnitude of postprandial glucose excursions and has the potential to be an adjunct treatment for diabetes and inflammatory-related diseases. The present investigation is aimed at understanding the molecular mechanism of action of PLG-007 and its galactomannan (GM) components GMα and GMβ (in a 1:4 mass ratio, respectively) on enzyme (i.e., α-amylase, maltase, and lactase) hydrolysis of glucose polymers using colorimetric assays and ^13^C HSQC NMR spectroscopy. The starch–iodine colorimetric assay indicated that GMα strongly inhibits α-amylase activity (~16-fold more potent than GMβ) and thus is the primary active component in PLG-007. ^13^C HSQC experiments, used to follow the α-amylase-mediated hydrolysis of starch and amylopectin, further demonstrate the α-amylase inhibitory effect of GMα via α-amylase-mediated hydrolysis of starch and amylopectin. Maltohexaose (MT6) was used to circumvent the relative kinetic complexity of starch/amylopectin degradation in Michaelis–Menten analyses. The V_max_, K_M_, and K_i_ parameters were determined using peak volume integrals from ^13^C HSQC NMR spectra. In the presence of PLG-007 with α-amylase and MT6, the increase in K_M_ from 7.5 ± 0.6 × 10^−3^ M (control) to 21 ± 1.4 × 10^−3^ M, with no significant change in V_max_, indicates that PLG-007 is a competitive inhibitor of α-amylase. Using K_M_ values, K_i_ was estimated to be 2.1 ± 0.9 × 10^−6^ M; however, the microscopic K_i_ value of GMα is expected to be larger as the binding stoichiometry is likely to be greater than 1:1. Colorimetric assays also demonstrated that GMα is a competitive inhibitor of the enzymes maltase and lactase. Overall, this study provides insight as to how PLG-007 (GMα) is likely to function in vivo.

## 1. Introduction

Starch, an α(1→4)-linked polymer of glucose (Glc), is the major complex carbohydrate found in food such as bread, potatoes, and rice. During digestion, starch is hydrolyzed by α-amylase to various smaller polysaccharides (e.g., dextrin), then into small oligosaccharides (e.g., maltohexaose, maltotriose, and maltose), and then with other hydrolases to the monosaccharide glucose. α-Amylase, found primarily in saliva and pancreas [1], randomly cleaves α(1→4) glycosidic linkages of starch and amylopectin via a double displacement mechanism with retention of an anomeric configuration. Amylopectin comprises of about 70% starch. Whereas starch is a linear polymer that forms a super-helix, amylopectin is a highly branched poly-glucose.

Maltase (α-glucosidase) hydrolyzes disaccharide maltose (Glc-α(1→4)-Glc) into two glucose molecules [2]. This enzyme is also known as sucrase because it can digest other dietary carbohydrates such as sucrose or table sugar (Glc-α(1→4)-Frc)) [3]. Sucrase is often found to be associated with isomaltase, and the two enzymes can form heterodimers, depending on the experimental conditions [4]. Lactase (also known as lactase-phlorizin hydrolase), another digestive enzyme of the β-galactosidase family, hydrolyzes the disaccharide lactose or milk sugar (Gal-β(1→4)-Glc) into galactose and glucose [5,6]. For diabetics, these enzymes are of particular interest because their activities eventually result in the production of glucose from ingested starch and amylopectin.

Increases in postprandial blood glucose excursion often occur as a result of sugar digestion. Diabetic patients require hypoglycemic drugs, such as insulin, to adequately control their blood sugar levels to reduce hyperglycemia associated with the ingestion of carbohydrates or food with a high glycemic index. Screening and diagnostic tests have been used to assess high levels of glucose in plasma and serum; e.g., Hera et al. [7] developed an approach to detect gestational diabetes mellitus using different neural network models and patient data. However, the efficacy of the diabetes management program is often limited due to non-compliance and poor uptake of systemic drugs, as well as the safety concern associated with systemic drug exposure. As such, the rising burden of type 2 diabetes mellitus and its associated morbidity and mortality remain a global epidemic problem. Thus, the introduction of a non-systemic compound would be a universal healthcare ideal in helping patients manage or maintain lower blood glucose levels without the risk of hypoglycemia. Acarbose [8,9] and Voglibose [10] are two anti-diabetic drugs already in clinical use to treat type 2 diabetes mellitus. Acarbose, an *a*-glucosidase inhibitor, has been intensively studied over the past decades. This compound is a natural microbial pseudotetrasaccharide and works by binding reversibly and competitively to the oligosaccharide binding site of *a*-glucosidases, leading to a reduction in the rate of digestion (hydrolysis) and absorption of glucose. This immediate effect allows avoidance of a postprandial glucose spike and reduction in the risk of hyperglycemia. However, adverse effects, including diarrhea, flatulence, and, in some cases, hepatitis [11], are often associated with the use of Acarbose; thus, alternative agents need to be explored and developed.

PLG-007 is an alternative developmental agent designed to reduce postprandial glucose excursions. PLG-007 is a proprietary combination of two plant-derived galactomannans (GMα and GMβ) [12,13]. GMα and GMβ have a β(1→4) mannan backbone, with a high density of α(1→6) linked galactose residues [12,13] with Man:Gal ratios of 1:1 and 2:1, respectively [14]. The PLG-007 formulation consists of GMα and GMβ mixed in a 1:4 GMα:β mass ratio. Several studies have demonstrated that leguminous seeds (and respective gums) can ameliorate metabolic symptoms associated with type 1 and type 2 diabetes in both humans and animals by reducing serum glucose and improving glucose tolerance [15,16], suggesting that the GM components in PLG-007 may play a part in glucose metabolism and may be effective in maintaining blood glucose levels.

The apparent blood glucose lowering effect of PLG-007 is effective in lowering postprandial hyperinsulinemia and may play a role in the regulation of lipid metabolism, thereby reducing the risks of cardiovascular disease and other comorbidities associated with type 2 diabetes mellitus. In an earlier open-label study of 24 patients with type 2 diabetes mellitus, 8 g and 16 g PLG-007 taken before a test meal reduced the 3-h post-prandial glucose area-under-curve (AUC) in 75% of patients [17,18]. The primary adverse events reported in that study were increased flatulence and bloating. In another proof-of-concept study in high-risk Chinese adults with prediabetes, the glycemic efficacy, tolerability, and safety of PLG-007 compared with the placebo were examined. The results indicated that low-dose PLG-007 (4 g, three times daily) attenuated a post-prandial rise in blood glucose with modest weight loss. Significant attenuation in several glycemic variability parameters, as measured by continuous glucose monitoring (CGM), were also observed in subjects receiving low-dose PLG-007. The compound was generally well tolerated and has the potential to be used as an adjunct to lifestyle modification in high-risk individuals for preventing diabetes [17].

In this study, we hypothesized that PLG-007 works by competitively inhibiting the activity of carbohydrate hydrolyzing enzymes involved in glucose metabolism, thereby diminishing release of glucose into the bloodstream and reducing glucose absorption. Studies using colorimetric assays and NMR spectroscopy were designed to investigate whether PLG-007 and its galactomannan components (GMα and GMβ) attenuate the enzymatic activities of α-amylase, maltase, and lactase. Results demonstrate that whereas both GMα and GMβ contribute to the inhibitory activity, GMα is the more active component and is the primary functional ingredient in PLG-007, functioning as a competitive inhibitor. Collectively, our data provide insight into how PLG-007 may function in vivo and act as a therapeutic agent for diabetic and inflammatory diseases.

## 2. Results

**Inhibition of α-amylase.** A starch–iodine assay was used initially to demonstrate whether the two galactomannans (GM) in PLG-007 (GMα and GMβ) could attenuate the activity of α-amylase. Table 1 shows the concentration-dependent changes from GMα and GMβ on the rate of porcine pancreatic α-amylase (PPA)-mediated starch hydrolysis (mg of starch hydrolyzed per minute). Of particular note, GMα attenuated the rate of starch hydrolysis (1 mg/mL) from 1.3 ± 0.26 mg/min at 0.5 mg/mL to 0.48 ± 0.23 mg/min at 4 mg/mL. Although GMβ also has an inhibitory effect on the enzyme, the magnitude was significantly less compared to GMα (Table 1). At a concentration of 16 mg/mL, the rate of amylase-mediated hydrolysis by GMβ was only 0.76 ± 0.09 mg/min. Similar results were obtained using human pancreatic α-amylase (HPA) and human salivary α-amylase (HSA) (Table 1). This was not surprising as both human and porcine sequences and structures (in particular around their active sites) are essentially the same [19,20]. On the other hand, Table 1 does show that with PPA (not HPA or HSA) there is a higher inhibitory effect of GM when incubated with a higher concentration of starch (5 mg/mL). Even though fractional differences in inhibition at 2 and 4 mg/mL are not as much as at 0.5 and 1.0 mg/mL, they are significant at the lower concentrations. It is unclear why this is so, but the trend may be related to the fact that starch can form supermolecular structures, especially at 5 mg/mL vis-à-vis 1 mg/mL, and the addition of another polysaccharide (GMα in this instance) may affect that structure, thus modulating activity.

**Figure 1:**
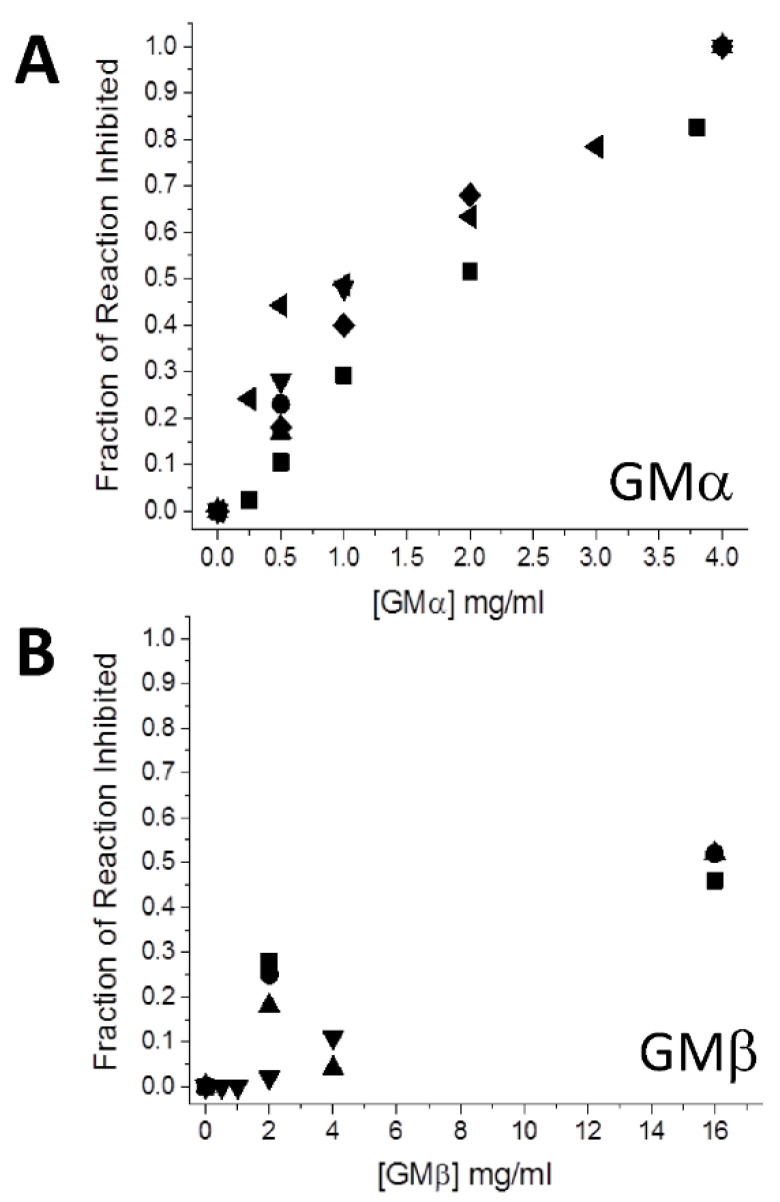
Colorimetric starch–iodine assay. The effectiveness of GMα (**A**) and GMβ (**B**) to inhibit starch hydrolysis (1 mg/mL) mediated by porcine pancreatic α-amylase (1 μM) is shown as a function of the concentration of GMα and GMβ. The assay was performed essentially as reported by Xiao et al. [21]. A total of 100 µL of 1 mg/mL (or 5 mg/mL) of soluble starch ± various concentrations (0.5 mg/mL up to 16 mg/mL) of GMα or GMβ were incubated for 20 min at 30 °C ± 5 µL enzyme, for a total concentration of 1 µM amylase/well. Assays were performed at 0.5, 1, 2, and 4 mg/mL of the GMs, Acarbose at 400 μM, and starch at 1 mg/mL or 5 mg/mL. At the end of the reaction time, the absorbance was read at 562 nm. The fraction of the reaction inhibited was calculated using U/mL values determined from the iodine–starch assay as described by Xiao et al. [21], with U/mL = (A_562_ control–A_562_ sample)/(A_562_ starch × 20 min × 0.1 mL reaction volume), a value that can be interpreted as mg of starch hydrolyzed per minute. U/mL values shown are averages of 4 or 5 separate experiments, each done in triplicate. Solution conditions are 20 mM potassium phosphate, pH 7, 30 °C.

Figure 1 plots the fraction of reaction inhibition (rate with GM divided by rate without GM) vs. the concentration of GMα (Figure 1A) and GMβ (Figure 1B), and Table 1 gives the average values of the individual data points shown in Figure 1. Results indicate that GMα is about 10-fold more effective than GMβ. Because the composition of GMα and GMβ in PLG-007 is in a 1:4 mass ratio, respectively, the corresponding ratio (0.5 mg GMα + 2.0 mg GMβ) was used in the assay (Table 1). The fraction inhibited (0.45) was nearly the same as that using the sum of 0.5 mg GMβ (0.3) and 2.0 mg GMβ (0.09). The same level of inhibition was also observed when an equivalent mass of GMα and GMβ from PLG-007 tablets was used. This finding also suggests that the sorbitol (~60% or ~2300 mg/4 g tablet) present in the PLG-007 formulation has no effect on α-amylase activity.

Galactomannans extracted from fenugreek seeds are also known to contain the co-isolate 4-hydroxy-isoleucine (4-OH-Ile), which itself has been found to inhibit glucose production [22]. NMR spectrometry was used to quantify the amount of 4-OH-Ile present in GMα. ^1^H NMR spectra of 4-OH-Ile at concentrations of 1 μM, 10 μM, and 100 μM were acquired and resonance intensities with that from GMα at 2 mg/mL were compared (data not shown). Results indicated that 1 mg/mL sample of GMα contained about 20 μM of 4-OH-Ile. At 16 mg/mL, GMβ contained no detectable amount of 4-OH-Ile. 4-OH-Ile at 50 μM, 100 μM, and 500 μM were assessed to examine the inhibitory effect of maltase-mediated hydrolysis of PNPG. Results show that 4-OH-Ile at these concentrations has no effect, consistent with previous findings that 4-OH-Ile is only active at higher concentrations [22]. This finding also indicates that the presence of 4-OH-Ile in PLG-007 does not contribute to its efficacy.

Based on the activity profile from Acarbose, a therapeutic agent being used to inhibit amylase-mediated activity in type 2 diabetic patients [23], the activity of GMα at a concentration of 2 mg/mL (~2 μM using a mass of 1 × 10^6^ Da) was calculated to be comparable with Acarbose at 400 μM (Table 1). However, since Acarbose is a small pseudotetrasaccharide with a 1:1 binding stoichiometry with α-amylase, it is assumed that the binding stoichiometry will be much greater with PLG-007 since GMα is a much larger polysaccharide. For example, if GMα has 20 binding sites, then the actual microscopic effect from GMα would be comparable at ~400 μM. This number of binding sites is reasonable given an analogous situation with another galactomannan that binds galectins with a galectin:GM binding stoichiometry of ~15–20:1 [24,25,26].

**α-Amylase-mediated hydrolysis of starch and amylopectin using ^13^C HSQC NMR.** Starch and amylopectin are polymers of α-glucose and are primarily hydrolyzed into primarily maltohexaose (MT6), maltotriose (MT3), and maltose (MT2) by α-amylase. One mole of MT2, for example, can then be hydrolyzed by maltase into two moles of glucose (Glc). To follow production of MT6, MT3, and MT2, the kinetics of α-amylase-mediated starch (and amylopectin) degradation were monitored using natural abundance ^13^C-^1^H HSQC spectra. Although ^1^H and ^13^C chemical shift assignments for MT6, MT3, and MT2 have been reported [27], resonance assignments were confirmed here by analyzing ^13^C-^1^H HSQC and HMBC spectra (data not shown).

**Figure 2:**
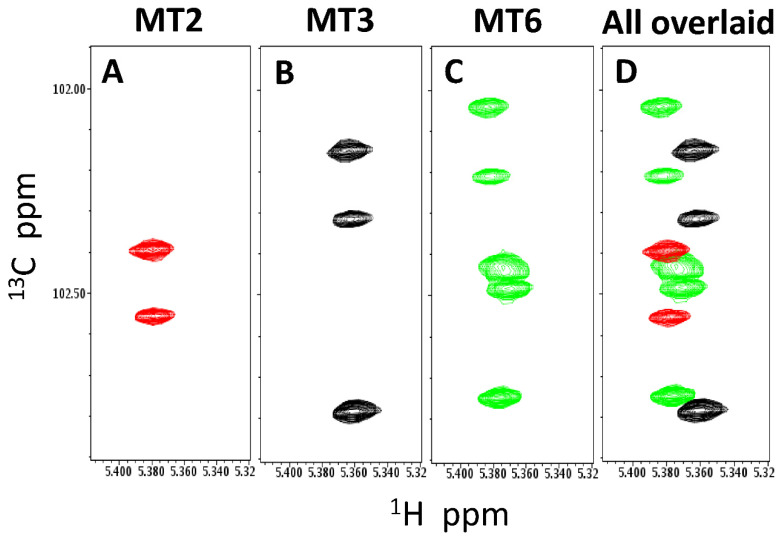
Natural abundance ^13^C HSQC spectra are shown for the standard saccharides maltose (MT2) (**A**), maltotriose (MT3) (**B**), and maltohexaose (MT6) (**C**). These ^13^C HSQC spectra were focused on acetal/anomeric groups and acquired over a narrow spectral window in the ^13^C dimension, effectively reducing the ^13^C sweep width producing folded spectra [28]. Because spectra are focused on acetal/anomeric groups, MT2 will show up as 2 peaks, MT3 as 3 peaks, and MT6 as 6 peaks with some overlap primarily for non-terminal glucose residues. Resonance assignments for MT2, MT3, and MT6 were reported by Goffin et al. (2009) and confirmed here by running and analyzing ^1^H-^13^C HSQC and ^1^H-^13^C HMBC experiments. (**D**) ^13^C HSQC spectra from panels (**A**–**C**) are superimposed in (**D**). Peaks are colored as red for MT2, black for MT3, and green for MT6. Solution conditions are 20 mM potassium phosphate, pH 7, 30 °C.

In order to acquire HSQC spectra more rapidly during kinetics runs, ^13^C HSQC spectra were acquired over a narrow spectral window in the ^13^C dimension. This effectively reduced the ^13^C sweep width, allowing for a more rapid acquisition of HSQC spectra at about 3 min per spectrum [28]. In addition, a greater signal intensity was obtained via acquisition of HSQC spectra for 10 min per time point. Figure 2A–C shows resonances obtained by running spectra on standard samples of MT6, MT3, and MT2. Figure 2D shows the overlay of all three individual spectra to illustrate how these maltose-based products from starch and amylopectin hydrolysis appear in the acetal-focused ^13^C HSQC spectra, with differences in resonance linewidths due to size changes and differences in chemical shifts due to environmental factors. Enzyme and substrate concentrations were also optimized to promote slower hydrolysis in order to acquire more time points during each kinetics run.

**Figure 3:**
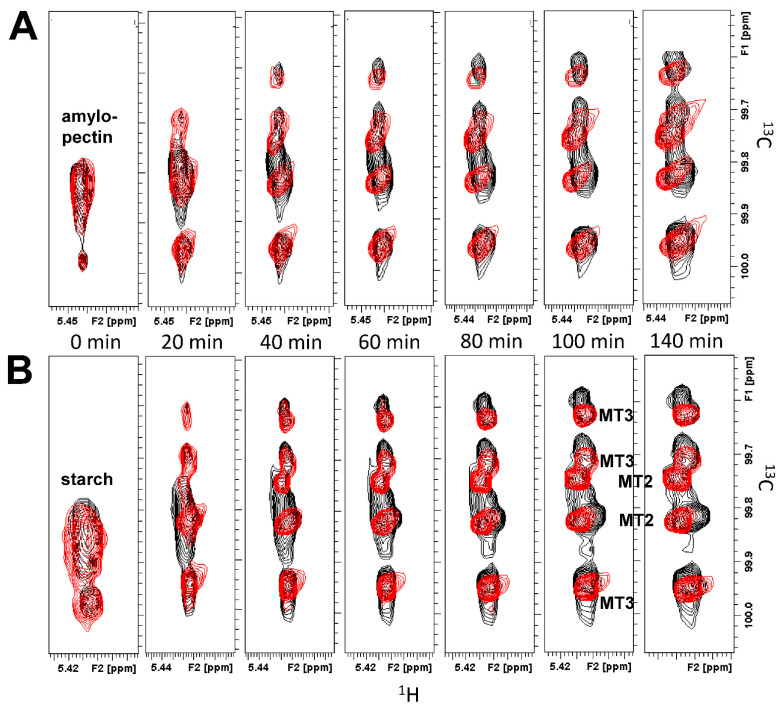
Natural abundance ^13^C HSQC showing time dependence of α-amylase-mediated (0.4 U/mL) hydrolysis of amylopectin (**A**) and starch (**B**). These kinetics runs focused on resonances from acetal groups and acquired ^13^C HSQC spectra over a narrow spectral window in the ^13^C dimension, as discussed in the text and legend to Figure 2. HSQC spectra are shown in the absence (black contours) and presence (red contours) of GMα (4.2 mg/mL). Time points are indicated on the figure. Solution conditions are 20 mM potassium phosphate, pH 7, 30 °C.

^13^C HSQC spectra of the anomeric resonance region, overlaid for amylopectin or starch (5 mg/mL) in the absence (black peaks) and presence (red peaks) of GMα (4.2 mg/mL) as a function of time, are shown in Figure 3. As hydrolysis proceeded, original resonances arising from amylopectin (or starch) disappeared as new peaks associated with MT2, MT3, and MT6 were formed. The time at which each HSQC spectrum was acquired is indicated, with only some of the time points acquired being shown. Initially, the presence of GMα resulted in a slightly higher level of MT2/MT3 production. However, by the end of a run, the amount of MT2/MT3 was significantly reduced compared to amylopectin (or starch) hydrolysis in the absence of GMα.

**Figure 4:**
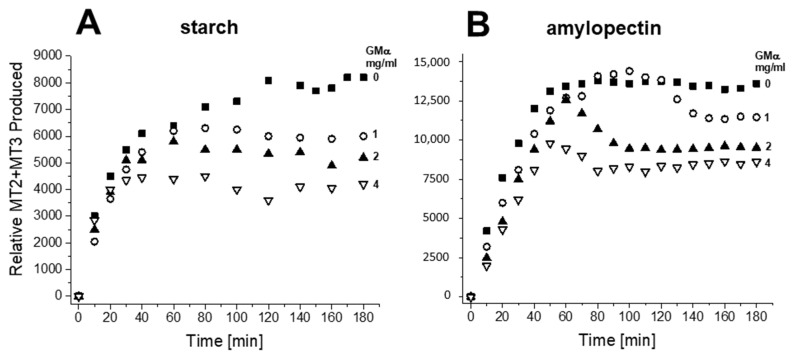
Using the ^13^C HSQC spectra as shown in Figure 3, the peak volume integrals were determined and plotted here as the net of MT2 and MT3 as a function of the time of α-amylase-mediated (0.4 U/mL) hydrolysis of starch (**A**) and amylopectin (**B**). Data are shown for the kinetics run performed in the absence (filled squares) and presence of GMα at concentrations of 1 mg/mL (open circles), 2.1 mg/mL (filled triangles), and 4.2 mg/mL (inverted open triangles). Solution conditions are 20 mM potassium phosphate, pH 7, 30 °C.

To quantify these data, volume integrals of resonances associated with MT2 + MT3 (total peak volumes) were plotted over time in Figure 4. The integral values shown on the Y-axis were proportional to the concentration of MT2 + MT3 produced. Due to the complexity of starch and amylopectin degradation (i.e., line broadening and unknown stoichiometry of degradation), the actual concentrations could not be determined. During the initial time period up to about 40 min, however, slopes were linear, as were expected for zeroth order kinetics where the substrate concentration is much greater than that of the enzyme. In the absence of GMα, production plateaus as time proceeds. However, in the presence of GMα, the kinetics are more complicated, with production levels appearing to peak and then decrease prior to leveling off. Although more data would be required to confirm this behavior, it is likely related to the interaction between GMα and amylase and/or starch or amylopectin. Nevertheless, GMα, in a concentration-dependent manner, effectively reduced the net production of MT2 + MT3 from both starch and amylopectin substrates. Moreover, the initial rate of production of MT2 + MT3 also decreased in the presence of GMα, in a concentration-dependent manner. The apparent initial rate of hydrolysis was derived by fitting initial slopes with linear curves. With starch as substrate, the initial rate goes from 300 ± 43 min^−1^ in the absence of GMα to 200 ± 23 min^−1^ in the presence of GMα. With amylopectin, results are the same with the initial rate going from 296 ± 26 min^−1^ in the absence of GMα to 204 ± 4 min^−1^ in the presence of GMα. Collectively, these results demonstrate that GMα effectively inhibits α-amylase-mediated hydrolysis of starch and amylopectin.

As mentioned above, resonance broadening from these relatively large polysaccharides did not always allow MT2, MT3, and MT6 products to be individually and/or accurately determined. GMα in particular has an average molecular weight of ~1000–2000 kDa, which, in turn, increases solution viscosity and resonance linewidths. Supplementary Figure S1 plots the measured viscosity (centi-Poise, cP) as a function of GMα and GMβ concentration. While there is little change in viscosity with increased GMβ concentration, a large change is observed in the presence of GMα. At 5 mg/mL, the viscosity of GMα is about 10 cP. However, at a 1:4 mass ratio of GMα to GMβ, as present in the PLG-007 formulation, the change in viscosity is greatly reduced (Supplementary Figure S1). Additional ^13^C HSQC spectral analyses with PLG-007 were performed to confirm this observation.

**Figure 5:**
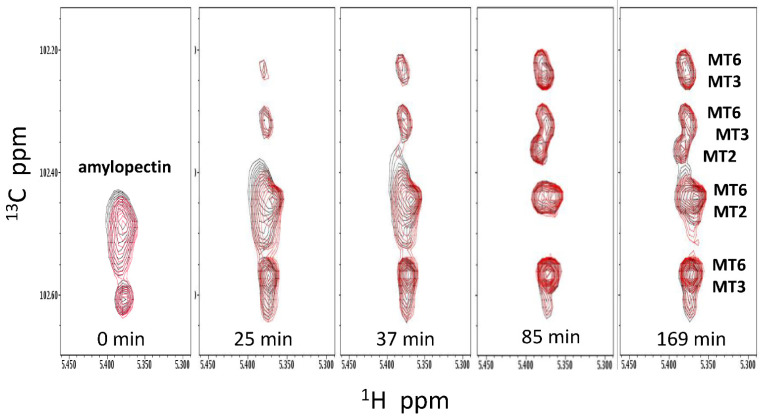
Natural abundance ^13^C HSQC spectra (focused on acetal resonances) showing the time dependence of α-amylase-mediated (0.4 U/mL) hydrolysis of amylopectin in the absence (black contours) and presence (red contours) of PLG-007 (4.2 mg/mL). Resonances are labeled for maltose (MT2), maltotriose (MT3), and maltohexaose (MT6), as discussed in the text. Time points are indicated on the figure. Solution conditions are 20 mM potassium phosphate, pH 7, 30 °C.

**Figure 6:**
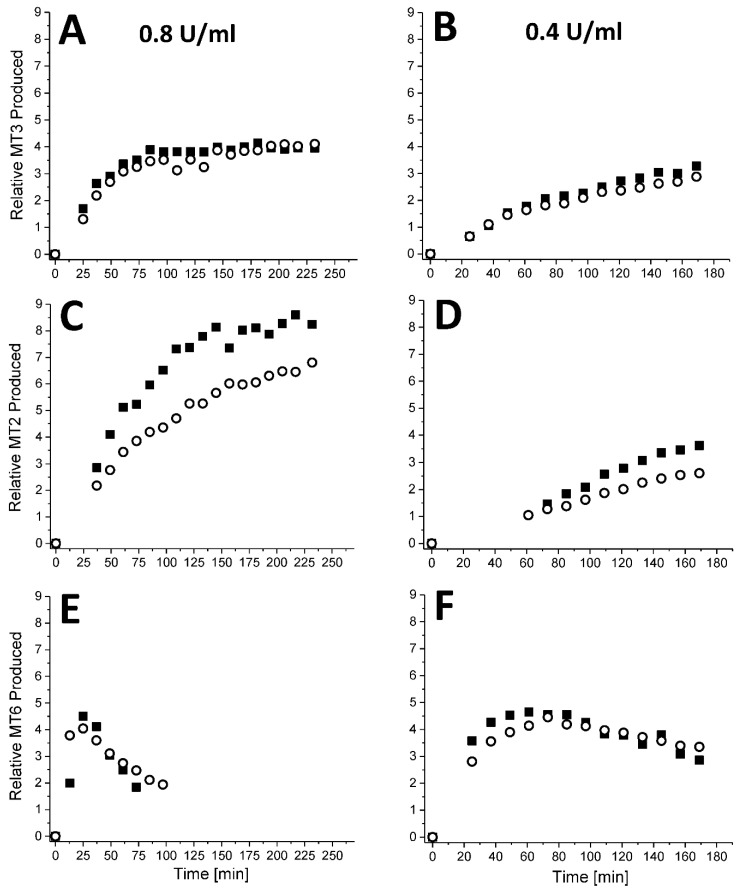
Using the ^13^C HSQC spectra as shown in Figure 5, peak volume integrals were determined and plotted individually for MT3 (**A**,**B**), MT2 (**C**,**D**), and MT6 (**E**,**F**) as a function of the time of hydrolysis of amylopectin mediated by α-amylase at 0.8 U/mL (**A**,**C**,**E**) and 0.4 U/mL (**B**,**D**,**F**). Data are shown for kinetic runs performed in the absence (filled squares) and presence of PLG-007 at a concentration of 4.2 mg/mL (open circles). Solution conditions are 20 mM potassium phosphate, pH 7, 30 °C.

Figure 5 shows ^13^C-folded HSQC spectra overlays of amylopectin in the absence (black peaks) and presence (red peaks) of PLG-007 at an equivalent GMα concentration. Because the spectral line widths are considerably narrower compared to those with GMα alone, the resonance intensities for MT6, MT3, and MT 2 could now be determined. The time course of hydrolysis for individual species (MT3, MT2, and MT6) is shown in Figure 6 for α-amylase concentrations of 0.8 U/mL (Figure 6A,C,E) and 0.4 U/mL (Figure 6B,D,F). At either enzyme concentration, production of MT3 (Figure 6A,B) was the same in the absence and presence of PLG-007, whereas production of MT2 (Figure 6C,D) was significantly reduced in the presence of PLG-007. On the other hand, MT6 levels rose relatively rapidly before dropping off. This is expected for a serial reaction in which the initial production of MT6 became the substrate for further hydrolysis to MT2. The presence of PLG-007 increased the levels of MT6 at the end of the kinetic runs, suggesting that PLG-007 interacts with amylase to compete with MT6 (substrate), while reducing the production of MT2 (Figure 6C,D). In situ, this could indicate that less MT2 will be available for hydrolysis by maltase and thus less glucose will be produced. It is hypothesized that PLG-007 may selectively affect the production of substrates (MT6 and MT2) in amylase-mediated hydrolysis of amylopectin.

**Figure 7:**
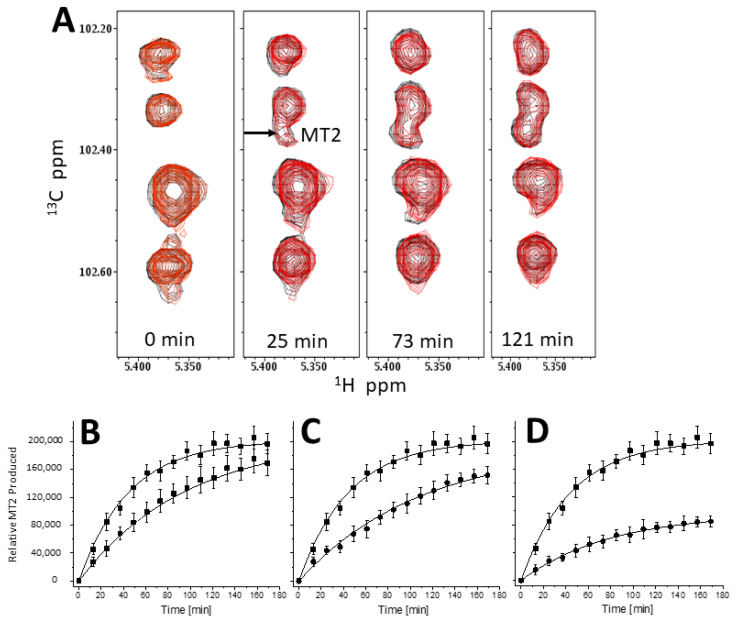
Natural abundance of ^13^C HSQC showing the time dependence of α-amylase-mediated (0.4 U/mL) hydrolysis of MT6 (**A**). Kinetics runs focused on resonances from acetal/anomeric groups. Resonance assignments for maltose (MT2), maltotriose (MT3), and maltohexaose (MT6) were reported by Goffin et al. [27], and confirmed here by running and analyzing ^1^H-^13^C HSQC and ^1^H-^13^C HMBC experiments. ^13^C HSQC spectra are shown in the absence (black contours) and presence (red contours) of PLG-007 (4.2 mg/mL). Time points are indicated on the figure. Using the full set of ^13^C HSQC spectra, peak volume integrals were determined for the best resolved resonance; i.e., MT2 as labeled, and plotted for production of MT2 at PLG-007 concentrations of 1.1 mg/mL (**B**), 2.1 mg/mL. (**C**), and 4.2 mg/mL (**D**). Data are shown for kinetic runs performed in the absence (filled squares) and presence of PLG-007 (open circles). Solution conditions are 20 mM potassium phosphate, pH 7, 30 °C.

**Michaelis–Menten analysis with α-amylase and substrate MT6.** α-Amylase-mediated (0.4 U/mL) hydrolysis of MT6 as substrate was examined using Michaelis–Menten analyses. Figure 7A shows the ^13^C HSQC spectra of MT6 alone (black peaks) and in the presence of PLG-007 (4.2 mg/mL, red peaks) acquired at several time points during the kinetics run. HSQC spectra were acquired every 10 min for a time span of 170 min or 180 min. The products of MT6 hydrolysis are MT2 and MT3, with MT2 showing the most resolved resonance. The MT2 peak is absent at the beginning of the kinetic run and only appears as hydrolysis proceeds. As there is no overlap with other resonances, the MT2 peak was considered the critical indicator for our investigation.

The relative amount of MT2 produced (volume integrals, average of three experiments ± SD) is shown as a function of time in Figure 7 in the absence and presence of PLG-007 at concentrations: 1.1 mg/mL (Figure 7B), 2.1 mg/mL (Figure 7C), and 4.2 mg/mL (Figure 7D). As the concentration of PLG-007 increases, the rate of production of MT2 attenuates. Initial rates of hydrolysis were determined by linear fits to the initial linear part of these kinetics curves. In the absence of PLG-007, the initial rate of α-amylase-mediated hydrolysis of MT6 to MT2 is 2513 ± 182 min^−1^. In the presence of PLG-007, initial rates decrease from 1602 ± 98 min^−1^ at 1.1 mg/mL to 1169 ± 141 min^−1^ at 2.1 mg/mL, and then to 796 ± 64 min^−1^ at 4.2 mg/mL. Results indicated that PLG-007 inhibits α-amylase activity in a concentration-dependent manner. The presence of PLG-007 at 2.1 mg/mL, and more so at 4.2 mg/mL, significantly (R^2^ = 0.99) attenuates MT2 production.

**Figure 8:**
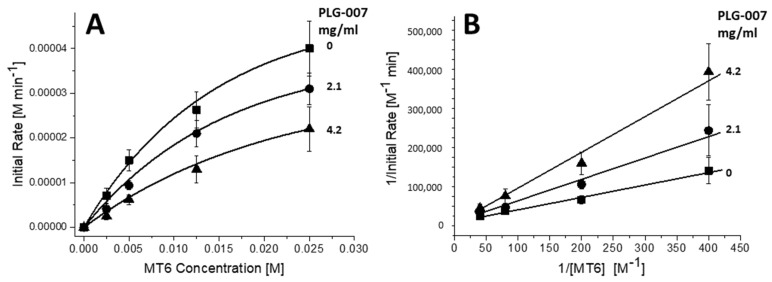
(**A**) Michaelis–Menten plots (initial rates of MT2 production vs. MT6 substrate concentration) are shown for the α-amylase-mediated hydrolysis of MT6 in the absence (filled squares) and presence of PLG-007 at 2.1 mg/mL (filled circles) and 4.2 mg/mL (filled triangles). (**B**) Using data shown in (**A**), Lineweaver–Burk plots of the inverse of the initial rates of MT2 production (i.e., hydrolysis) vs. the inverse of the MT6 substrate concentration, are shown in the absence (filled squares) and presence of PLG-007 at 2.1 mg/mL (filled circles) and 4.2 mg/mL (filled triangles). The concentration of MT2 produced was determined by using a calibration curve generated by acquiring NMR spectra of maltose (MT2) at known concentrations. The slope of each curve provides a measure of K_M_/V_max_ and the Y-intercept gives 1/V_max_, as discussed in the text. General solution conditions are 20 mM potassium phosphate, pH 7, 30 °C. Error bars indicate the standard deviations of three different experiments, each run in duplicate.

Since volume integrals are proportional to actual concentrations, conversion to molar concentrations of MT2 based on the initial and final concentrations of substrate MT6 were performed. In Figure 8A, Michaelis–Menten plots showed initial rates (M min^−1^) vs. the concentration (M) of MT6. Lineweaver–Burk plots (Figure 8B) provided insight into the mechanism of action of PLG-007. The slopes of these plots yielded K_M_/V_max_ and the Y-intercepts yielded 1/V_max_. Whereas V_max_ is invariable, K_M_ varies from 7.5 ± 0.6 × 10^−3^ M (without PLG-007), to 12 ± 0.83 × 10^−3^ M at 2.1 mg/mL PLG-007, and 21 ± 1.4 × 10^−3^ M at 4.2 mg/mL PLG-007. Essentially, the same results were found using GMα alone (data not shown). Since K_M_ is increased in the presence of PLG-007 and V_max_ does not appear to change significantly, we concluded that PLG-007 is a competitive inhibitor of α-amylase, and we speculate that the binding epitope on GMα interacts with the enzyme to block its active site and competitively inhibits the binding of the MT6 substrate.

From these Michaelis–Menten data, we also calculated the equilibrium inhibition constant, K_i_, using Equation (1):

K_M_^app^ = K_M_ (1 + [I]/K_i_) (1)

where K_M_ is the Michaelis–Menten constant for the enzyme in the absence of an inhibitor; K_M_^app^ is the change in K_M_ in the presence of an inhibitor; and [I] is the concentration of the inhibitor. Since the PLG-007 formulation is consisted of primarily GMα, GMβ, and sorbitol (which is inactive), the inhibitor concentration was calculated using the weight-averaged MWs of GMα and GMβ at a 1:4 GMα:GMα mass ratio (i.e., ~580 kDa). Moreover, with GMα being 10-fold more active than GMβ, the relative concentration was adjusted accordingly, and the effective inhibition concentration was calculated to be 2.9 μM and 1.5 μM. Using both values, K_i_ was estimated to be 2.1 ± 0.9 × 10^−6^ M. However, since GMα (and even the less potent GMβ) is likely to have a number of binding sites for α-amylase, the binding stoichiometry would be greater than 1:1; thus, the actual K_i_ value is expected to be larger.

**Figure 9:**
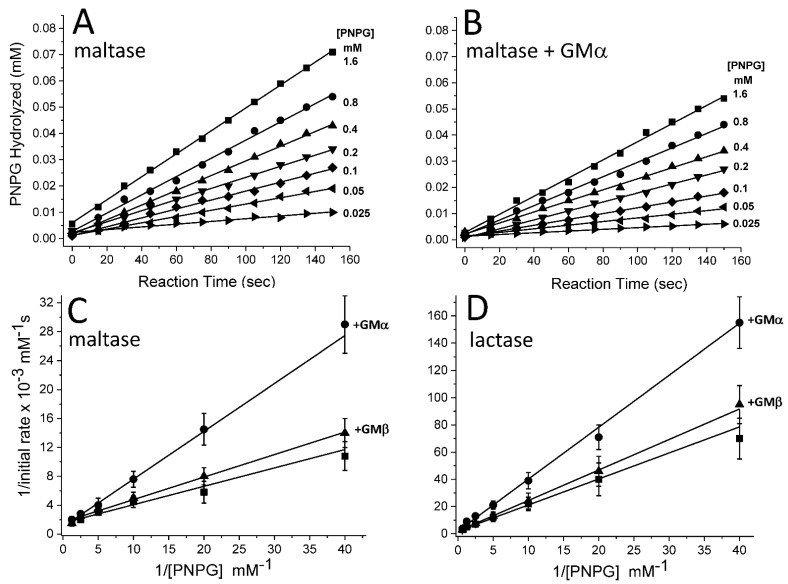
Colorimetric PNPG assays with maltase and lactase. (**A**) The amount of para-α-nitrophenyl-D-glucopyranoside (α-PNPG) hydrolyzed by maltase (1 unit) is shown for α-PNPG concentrations from 0.025 mM to 1.6 mM, as indicated in the figure. (**B**) The amount of α-PNPG hydrolyzed by maltase (1 unit) in the presence of GMα (2 mg/mL) is shown for α-PNPG concentrations from 0.025 mM to 1.6 mM, as indicated in the figure. Using initial rates of hydrolysis (e.g., those with maltase shown (**A**,**B**)), Lineweaver–Burk plots are shown for PNPG (para-α- or para-β-nitrophenyl-D-glucopyranoside) hydrolysis in the presence of maltase (**C**) and lactase (**D**) in the absence and presence of 2 mg/mL GMα or 8 mg/mL GMβ. From the analyses of these data, the V_max_ and K_M_ parameters are given in Table 2. Error bars indicate the standard deviations of 3 or 4 different experiments, each run in duplicate.

**Inhibitory effects on maltase and lactase.** Michaelis–Menten studies were performed in colorimetric PNPG assay to determine the V_max_ and K_M_ values for maltase and lactase acting on the appropriate PNPG substrate (i.e., para-α- or para-β-nitrophenyl-D-glucopyranoside). Figure 9A,B shows the kinetics data with maltase acquired at α-PNPG concentrations from 0.025 mM to 1.6 mM in the absence and presence of GMα. As expected, the initial rate (mM s^−1^) of hydrolysis is increased as the α-PNPG concentration is increased (Figure 9A) and the presence of GMα attenuated those rates (Figure 9B). Lineweaver–Burk plots of these data (Figure 9C) indicate that GMα (2 mg/mL) significantly attenuates the rate of hydrolysis, and GMβ shows no significant effect vis-à-vis control. The same trends were observed with the enzyme lactase (and its substrate β-PNPG) in the absence and presence of GMα and GMβ (Figure 9D).

Analyses of these Lineweaver–Burk plots yield V_max_ and K_M_ parameters (Table 2). Although GMα has no apparent effect on V_max_, it does have a significant effect on K_M_, in a concentration-dependent manner. Similar to α-amylase, GMα acts as a competitive inhibitor of maltase and lactase. Using Equation (1) and the average MW for GMα (1 × 10^6^ Da), the K_i_ value was estimated to be 3.3 × 10^−6^ M for lactase and 2 × 10^−6^ M for maltase. Consistent with α-amylase, the actual K_i_ values are anticipated to be larger, since the binding stoichiometry of the enzyme to GMα is likely to be greater than 1:1.

Effects from GMα alone (2 mg/mL) and in combination with GMβ (8 mg/mL) at the 1:4 mass ratio were also examined. While GMβ alone showed no effect on the kinetics (V_max_ or K_M_) of the maltase–mediated hydrolysis of α-PNPG, a small effect on K_M_ in the lactase–mediated hydrolysis of β-PNPG was observed, where K_M_ was increased from 0.18 (±0.03) mM to 0.24 (±0.04) mM in the presence of GMβ.

**Effects from solution viscosity on enzyme activity.** Since high solution viscosities can attenuate enzymatic activity [29,30,31], possible indirect effects on solution viscosity from GMα were also examined. Although GMβ or GMα:GMβ (1:4) has no great effect on solution viscosity, GMα alone at 4 mg/mL displays a solution viscosity of ~8 cP (Supplementary Figure S1). The effects of solution viscosity on enzyme activity was examined using the colorimetric assay with maltase to hydrolyze α-PNPG in the presence of 40% (*v*/*v*) and 60% (*v*/*v*) glycerol. At 30 °C, the aqueous solution viscosities with glycerol at these concentrations are 2.82 cP and 7.06 cP, respectively. Supplemental Figure S2 shows Lineweaver–Burk plots for the kinetics data acquired at 0%, 40%, and 60% glycerol. Although the hydrolytic activity of maltase was attenuated in response to increased glycerol viscosity, the effect is relatively small in comparison to the viscosity effect observed in the presence of 4 mg/mL GMα. Moreover, glycerol at these concentrations appeared to have a significant effect only on V_max_, which was decreased from 8.4 (±0.6) × 10^4^ mM s^−1^ to 4.6 (±0.5) × 10^4^ mM s^−1^ to 3.1 (±0.5) × 10^4^ mM s^−1^ at 0%, 40%, and 60% glycerol, respectively. K_M_ values, however, were not dependent on viscosity at concentrations of 0.22 (±0.04), 0.16 (±0.05), and 0.18 (±0.05), respectively. These V_max_ and K_M_ values are characteristic of attenuated enzyme activity mediated solely by increases in solution viscosity [29,30,31]. This finding is contrary to those values determined in the presence of GMα, where V_max_ was essentially unchanged and K_M_ was observed to increase with increasing amounts of GMα (Table 2). Collectively, these results suggest that although GMα may increase solution viscosity, its primary effect is to function as a competitive inhibitor of maltase (and by reasonable extension of α-amylase and lactase) activity. Moreover, the presence of GMβ in PLG-007 effectively negates the viscosity effects observed in the presence of GMα.

## 3. Discussion

In recent times, the field of pharmaceutical drug discovery has expanded in scope to include not only small molecules and antibodies, but many other therapeutic agents such as aptamers, packaged liposomes, and nanoparticles. These are composed primarily of proteins, nucleic acids, and/or lipids. On the other hand, the development of saccharides (especially large polysaccharides and those derived from plants) as therapeutic agents has not been the major focus in the pharmaceutical industry. PLG-007, a novel polysaccharide-based therapeutic, composed primarily of two large galactomannans (GMα and GMβ), may represent a new class of therapeutic agents that is effective in reducing glycemic variability via reduction (blunting) of postprandial glucose excursion. A recent study has demonstrated PLG-007′s ability to attenuate a post-prandial rise in blood glucose with modest weight loss [17]. Given the potential of this compound as an adjunct in a diabetes treatment regimen, the present study was designed to gain insight into the mechanism of action of PLG-007 (GMα and GMβ) at the molecular level against key carbohydrate-hydrolyzing enzymes (α-amylase, maltase, and lactase) that play significant roles in the digestion of glucose-containing polysaccharides.

In the present study, our results demonstrate that PLG-007 is an effective inhibitor of these enzymes, with GMα being the primary active component. PLG-007 (GMα) functions mechanistically as a competitive inhibitor, binding to these enzymes at or around their active sites to compete with natural substrate binding. Apparent K_i_ values of PLG-007 (GMα) for α-amylase with MT6 as substrate (or for maltase and lactase with substrates α-PNPG and β-PNPG, respectively) were found to be in the micromolar range. The presumed greater GMα binding stoichiometry is thought to enhance its avidity for these enzymes and thus increase its overall inhibitory potency. Due to the α-amylase-mediated production of multiple hydrolytic products with starch and amylopectin as substrates, Michaelis–Menten analyses could not be performed. Nevertheless, our data do provide evidence that PLG-007 (GMα) significantly attenuates enzyme-mediated hydrolysis. A burst in MT2 and MT3 production that plateaus off in a concentration-dependent manner in presence of GMα was observed when starch was used as substrate. On the other hand, an initial burst in MT6 production (that is further hydrolyzed to MT2 or MT3) was observed with amylopectin as substrate. It is also interesting to note that MT2 production was greatly inhibited in the presence of PLG-007 (GMα).

The resulting inhibitory effect from PLG-007 (GMα) was somewhat surprising since GM is not the normal glucose-based substrate for these carbohydrate-hydrolyzing enzymes. We speculate that this may be due to their structure that contain galactose in α(1→6)-linked side chains off of a polymannan backbone. Galactose is an anomer of glucose that differs only in the configuration of its 4-OH group, which is axial in galactose and equatorial in glucose. This structural similarity may allow galactose residues in GMα to interact in a similar manner as glucose in the enzyme’s active site. Whereas GMβ in the PLG-007 formulation is not as effective as GMα, it does function in concert with GMα by reducing GMα-induced solution viscosity, perhaps due to its ability to open up active site binding epitopes within GMα. In GMα, each mannose group within the β(1→4)-linked mannan backbone has one α(1→6)-branched galactose residue, in a Man:Gal ratio of 1:1. Therefore, it is conceivable that galactose residues in GMα are conformed in such a fashion to mimic the helical-like stretches of glucose residues found in starch or amylopectin. In GMβ, the Man:Gal ratio is higher, at 2:1, such that there are fewer galactose groups, which could explain the lower efficacy of GMβ compared to GMα.

The activity of PLG-007 (GMα) compares favorably with that of Acarbose, a natural microbial pseudotetrasaccharide presently being used to treat type 2 diabetes [23]. Acarbose is also a competitive inhibitor of α-glucosidases and binds α-amylase strongly with a K_d_ in the nanomolar range [32]. Although a higher amount of PLG-007 (GMα) would theoretically be required to reach a K_d_ of comparable magnitude, PLG-007 has demonstrated efficacy in reducing the magnitude of 2-h postprandial glucose excursions in individuals with type 2 diabetes. In another clinical study, Lee et al. [18] found that consumption of PLG-007 (6 to 12 g) prior to carbohydrate food or sugary beverage intake significantly reduced the postprandial glucose levels and insulin responses in a dose-dependent manner. In a third study, Luk et al. [17] reported that low-dose PLG-007 (4g, 3× daily) also attenuated a post-prandial rise in blood glucose and reduced body weight modestly. Interestingly, the PLG-007 doses (4g to 16g) used in these studies correspond to the calculated theoretical concentration (1.6 mg/mL to 3.2 mg/mL upon ingestion) in humans (blood volume at ~5 L). Nevertheless, it is possible that some galactomannans may move undigested through the GI track, and further studies are needed to investigate and confirm this proposal.

## 4. Methods and Materials

### 4.1. Enzymes and Polysaccharides

Human pancreatic α-amylase (HPA), human salivary α-amylase (HSA), and porcine pancreatic α-amylase (PPA), as well as porcine maltase and lactase, were purchased from Megazyme, Inc. Other enzymes, chemicals, and reagents were purchased from Sigma-Aldrich (St. Louis, MO, USA). PLG-007 and the galactomannans GMα and GMβ were provided by PharmaLectin, Inc., Boston, MA, USA.

### 4.2. Sample Preparations

All solutions, enzymes, and saccharides were prepared in an aqueous buffer: 20 mM potassium phosphate, 2 µM CaCl_2_, and 0.02% NaN_3_, pH 7. Enzymes were buffer exchanged six times using 10 kDa Amicon Ultra−0.5 mL centrifugal filters. Enzyme concentrations were determined using a NanoDrop 8000 UV-Vis Spectrophotometer and E1% A_280_, and solutions were diluted to 20 µM, aliquoted, and frozen until use. Starch and amylopectin were vortexed and used immediately or incubated at RT overnight.

The PLG-007 formulation consists of GMα and GMβ mixed in a 1:4 GMα:β mass ratio. These galactomannans have a 1,4-β-D-mannan backbone and 1,6-α-D-galactose side chains with Man/Gal mass ratios of 1:1 and 2:1, respectively. These polysaccharides are poly-dispersed with an average molecular weight ranging from ~1000 to 2000 kDa for GMα and ~200 kDa for GMβ. In aqueous solution, GMβ is highly soluble, whereas GMα only has a solubility limit of 5 mg/mL (0.5%). The solubility of GMα is improved significantly when it is mixed with GMβ in a 1:4 (GMα:GMβ) mass ratio, similar to the present PLG-007 formulation, where each 4 g tablet contain s 320 mg GMα, 1280 mg GMβ, and 2303.2 mg sorbitol. Since heating at high temperatures (above 50 °C) may promote decomposition of some galactomannans, stock solutions (50 mg in 50 mL of aqueous buffered solution) were prepared at RT (20 mM potassium phosphate, 2 µM CaCl_2_, and 0.02% NaN_3_, pH 6.9). The solution was then vortexed and allowed to stand for 2 days to obtain a clear solution. The solution was centrifuged at 5000 rpm for 20 min to remove any particulate matter prior to use.

### 4.3. Starch–Iodine Colorimetric Assay

The starch–iodine assay was modified from the methodology reported by Xiao et al. [21]. A total of 100 µL of 1 mg/mL (or 5 mg/mL) of soluble starch ± various concentrations (0.5 mg/mL up to 16 mg/mL) of GMα or GMβ were incubated for 20 min at 30 °C ± 5 µL enzyme, for a total concentration of 1 µM amylase/well. With Acarbose, the assay was performed at 400 μM. At the end of the reaction time, the absorbance was read at 562 nm. U/mL was calculated as (A_562_ control − A_562_ sample)/(A_562_ starch × 20 min × 0.1 mL reaction volume). Values reported in this study were averages of ~4 or 5 separate experiments (done in triplicates) with standard deviations.

### 4.4. Para-Nitrophenyl-Glucose (PNPG) Colorimetric Assay

To assess the effects of GMα and GMβ on maltase and lactase, a colorimetric assay with para-α (or -β)-nitrophenyl-D-glucopyranoside (PNPG, from Calbiochem) was used as substrate. Samples were prepared in 20 mM potassium phosphate, pH 7, in 96-well plates and a 30 °C water bath. To initiate the hydrolysis reaction, 50 μL of enzyme (4 U/mL) was added to a 5.5 mL reaction vessel, with 20 μL being removed every 15 s. The reaction was stopped by adding 80 μL of 200 mM NaCO_3_. Reactions were monitored as a function of time following the addition of an enzyme. A color change (A_405_) indicates initiation of enzyme-mediated hydrolysis of PNPG. A para-nitrophenol standard (Sigma Aldritch) ± GMα or GMβ was used to construct a standard curve. At the end of each kinetic run, a ThermoMax microplate reader was used to read the plates at 405 nm. The concentration of the cleaved substrate was derived using linear regression analysis of a standard curve.

Formation of product was followed in the absence and presence of GMα or GMβ at various substrate:GMα ratios. Initial reaction rates were determined from linear fits of the product concentration vs. time, where the slope yields the initial rate constant, *k*, in units of inverse time (min^−1^ or s^−1^). By repeating these experiments as a function of PNPG substrate concentration, Lineweaver–Burk plots (inverse of the initial rate (mM^−1^s) vs. the inverse of the α-PNPG concentration (mM^−1^)) were constructed and the V_max_ and K_m_ values were determined.

### 4.5. Viscosity Measurements

Viscosity was measured at RT on a TA Instruments AR-G2 rheometer with a concentric cylinder using bob and cup geometry. A total of 15 mL of GMα, GMβ, or a 1:4 molar combination was prepared in 20 mM potassium phosphate, 2 µM CaCl_2_, and 0.02% NaN_3_, pH 7. Strain and frequency sweeps were performed to define the linear viscoelastic regions. Subsequent oscillatory stepped flow procedures were undertaken with shear rates (1/s) from 1 to 100, 100 to 0.01, and finally 1 to 1000. Results were visualized using Rheology Advantage Data Analysis software. For reference, 1 Pa.s = 1000 cP, where “Pa” stands for Pascal, “s” for seconds, and “cP” for centiPoise.

### 4.6. NMR Spectroscopy

NMR experiments were performed at 30 °C (303 K) on Bruker Avance 600 MHz, 700 MHz, or 850 MHz spectrometers, each equipped with an H/C/N triple-resonance probe and *x*/*y*/*z* triple-axis pulse field gradient unit. Raw data were processed using NMRPipe [33] and analyzed using NMRview [34]. For ^1^H NMR experiments, 16 k data points were acquired with a sweep width of 15 ppm.

^13^C HSQC NMR spectra were acquired to follow the kinetics of polysaccharide hydrolysis. Even though ^1^H and ^13^C resonance assignments of products from α-amylase-mediated hydrolysis of starch and amylopectin (i.e., maltose (MT2), maltotriose (MT3), and maltohexaose (MT6)) have previously been reported [27], gradient sensitivity-enhanced versions of two-dimensional ^1^H-^13^C HSQC and ^1^H-^13^C HMBC experiments were performed to confirm assignments in this study. HSQC spectra were acquired as 408 (*t*1) × 2048 (*t*2) complex data points in the carbon and proton dimensions, respectively. HSQC spectra give correlations between ^1^H and ^13^C resonances within each sugar ring, and HMBC spectra give long-range correlations between/among ^1^H and ^13^C resonances within each sugar ring and between linked sugar rings.

To reduce the acquisition times during kinetic runs of enzyme-mediated sugar hydrolysis, resonances from acetal groups were examined and ^13^C HSQC spectra were acquired over a narrow spectral window in the ^13^C dimension to effectively reduce the ^13^C sweep width [28]. Resonances were readily identified using full HSQC spectra and running spectra on standard samples of MT6, MT3, and MT2. HSQC spectrums were acquired for 10 min per time point in order to obtain a greater signal intensity. In these NMR experiments, enzyme and substrate concentrations were also optimized to promote slower sugar hydrolysis in order to acquire more time points during each kinetic run.

NMR samples contained a 300 µL solution (20 mM potassium phosphate, 2 µM CaCl_2_, and 0.02% NaN_3_, pH 6.9) with 1–5 mg/mL soluble starch, amylopectin, or maltohexaose (MT6) and 10% D_2_O ± GMα, GMβ, 1:4 GMα:GMβ mass ratio, or PLG-007. In the presence of PLG-007, the concentration given was the GMα equivalent in mg/mL. In total, 10 µM 4,4-dimethyl-4-silapentane-1-sulfonic acid (DSS) was added to all solutions to calibrate the chemical shifts and to normalize the resonance intensities to determine the concentrations of hydrolyzed saccharides. ^1^H NMR spectra were acquired prior to addition of enzymes, and then enzymes (1 μM) were added to the NMR tube, and spectra were acquired as a function of time. Peak intensities were measured, normalized to DSS, and concentrations were determined by using a standard curve determined at known concentrations of maltose and maltotriose.

## 5. Conclusions

In conclusion, our results provide insight into how PLG-007 (a combination of galactomannans GMα:GMβ at a mass ratio of 1:4), as well as its individual components (GMα and GMβ), at the molecular level may exert effects in vivo to blunt postprandial glucose excursion and weight loss (secondary). Here, we demonstrate that GMα is highly active at inhibiting the α-amylases, maltase, and lactase that hydrolyze glucose-based saccharides. Moreover, GMα is more active than GMβ that is present in PLG-007 to reduce GMα-mediated solution viscosity and improves its interaction with these hydrolytic enzymes. Aside from using the standard colorimetric iodine–starch assay to demonstrate this, we also employed a novel fold-over HSQC NMR approach to derive the Michaelis–Menten kinetic parameters (K_M_ and V_max_) using the defined glucose-based substrates maltose (MT2), maltotriose (MT3), and maltohexaose (MT6). Given the ease of administration and high levels of tolerance, PLG-007 has the potential to be used in diabetes treatment and prevention programs. Future studies are required to test the feasibility and effectiveness of PLG-007 and confirm its clinical effectiveness in a larger healthcare or management regimen.

## Figures and Tables

**Table 1:** Rates of α-amylase-mediated hydrolysis of starch as measured by the starch–iodine assay and MT2/MT3 resonance intensity using NMR. Amylase-inhibitory potential to assess the effectiveness of GMα and GMβ on starch hydrolysis was performed essentially according to the protocol of Xiao et al. [21]. Although most assays were performed using porcine pancreatic α-amylase (PPA), a few were done using human pancreatic α-amylase (HPA), and human salivary α-amylase (HSA). A total of 100 µL of 1 mg/mL (or 5 mg/mL) of soluble starch ± various concentrations (0.5 mg/mL up to 16 mg/mL) of GMα or GMβ were incubated for 20 min at 30 °C ± 5 µL enzyme, for a total concentration of 1 µM amylase/well. The assay was also performed with 400 µM acarbose. At the end of the reaction, the absorbance was read at 562 nm. Assays were performed at 0.5, 1, 2, and 4 mg/mL, Acarbose at 400 µM, and starch at 1 mg/mL or 5 mg/mL. U/mL = (A_562_ control − A_562_ sample)/(A_562_ starch × 20 minutes × 0.1 mL reaction volume), and is interpreted to indicate X. U/mL values shown are averages of 4 or 5 separate experiments each done in triplicate.

		PPA		HPA	HAS
	Conc	U/mL	Fraction	Fraction	Fraction
	(mg/mL)	(mg/min)	Inhibited	Inhibited	Inhibited
**Starch**	1.0	1.86 ± 0.3	---		
**GMα:β**	2.5	1.02 ± 0.1	0.45	0.41	0.36
**1:4 ratio**					
**GMα**	0.5	1.3 ± 0.26	0.3		
	1.0	1.2 ± 0.16	0.35		
	2.0	0.72 ± 0.19	0.61	0.52	0.42
	4.0	0.48 ± 0.23	0.74		
**GMβ**	0.5	1.73 ± 0.69	0.07		
	1.0	1.85 ± 0.58	0.005		
	2.0	1.68 ± 0.3	0.09	0.05	0.05
	4.0	1.54 ± 0.47	0.17		
	16.0	0.76 ± 0.17	0.59		
**Acarbose**		0.38 ± 0.09	0.79		
**Starch**	5.0	1.45 ± 0.11			
**GMα**	0.25	1.1 ± 0.1	0.27		
	0.5	0.81 ± 0.26	0.44		
	1.0	0.74 ± 0.16	0.49		
	2.0	0.53 ± 0.18	0.64	0.58	0.49
	4.0	0.31 ± 0.19	0.78		

**Table 2:** Rates of enzyme-mediated hydrolysis of PNPG disaccharides.

	V_max_ (×10^4^ mM s^−1^)		K_M_ (mM)	
	PNPG	+GM	PNPG	+GM
**Maltase**	7.9 ± 0.8	8.6 ± 1	0.18 ± 0.05	0.52 ± 0.06
(GMα 2 mg/mL)				
**Maltase**	7.4 ± 0.8	7.8 ± 1	0.2 ± 0.04	0.19 ± 0.05
(GMβ 8 mg/mL)				
**Maltase**	8.2 ± 0.7	8.1 ± 0.8	0.22 ± 0.06	0.45 ± 0.08
(GMα 2 mg/mL				
+GMβ 8 mg/mL)				
**Lactase**	2.1 ± 0.3	1.9 ± 0.4	0.16 ± 0.04	0.36 ± 0.06
(GMα 2 mg/mL)				
**Lactase**	2.4 ± 0.5	2.5 ± 0.6	0.18 ± 0.03	0.24 ± 0.04
(GMβ 8 mg/mL)				
**Lactase**	2.5 ± 0.4	2.6 ± 0.5	0.17 ± 0.06	0.33 ± 0.05
(GMα 2 mg/mL +GMβ 8 mg/mL)				

## Data Availability

Data supporting reported results can be found at the Mayo Lab, University of Minnesota.

## Supplementary Materials

The following supporting information can be downloaded at: https://www.mdpi.com/article/10.3390/ijms23147739/s1.
