# Supplementary material for: PLG-007 and Its Active Component Galactomannan-α Competitively Inhibit Enzymes That Hydrolyze Glucose Polymers
Source: Int J Mol Sci. 2022 Jul 13;23(14):7739. doi: 10.3390/ijms23147739 (PMC9316267; doi:10.3390/ijms23147739)
Supplement: Supplementary file 1 [file ijms-23-07739-s001.zip › ijms-1809855-supplementary.pdf]

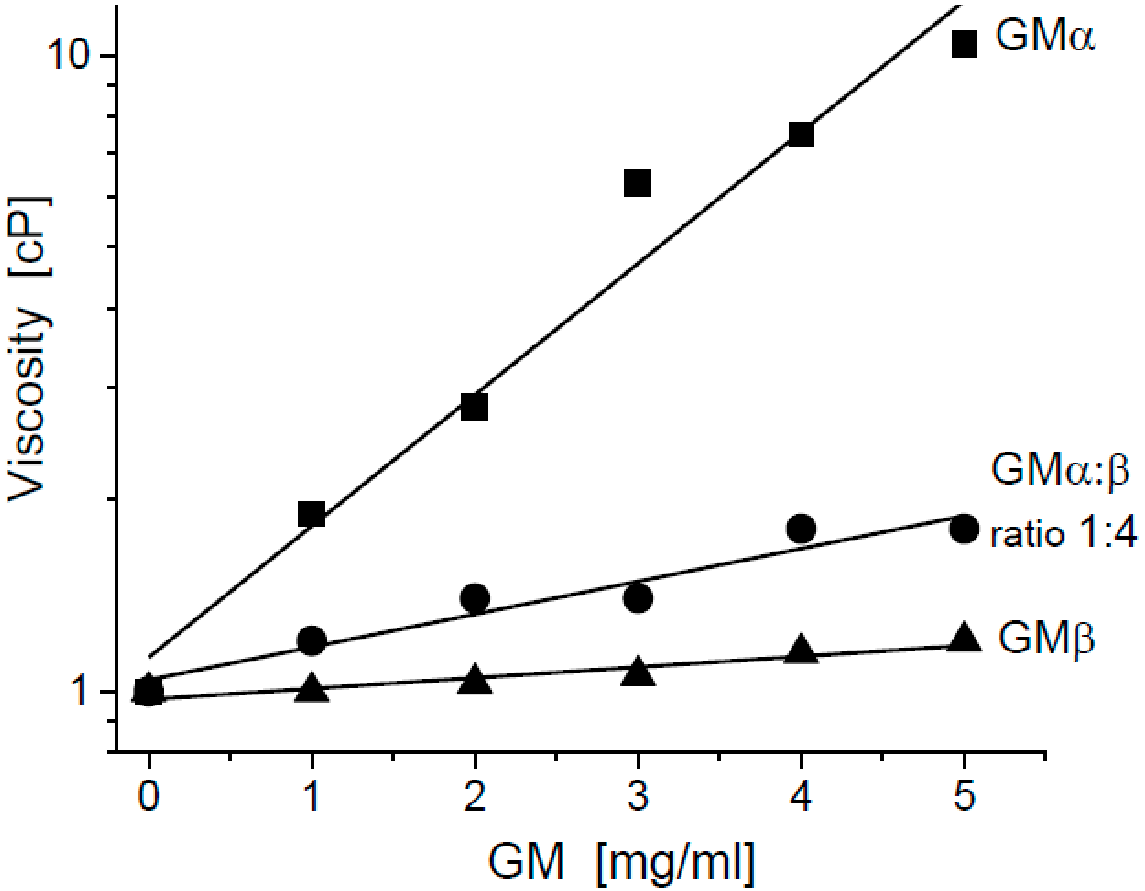

Supplemental Figure S1. The effect of GMα, GMβ, and a 1:4 molar ratio of GMα:GMβ on solution viscosity (cP, centi-Poise) is shown as a function of the GM concentration. For data on the 1:4 molar ratio of GMα:GMβ, the total concentration is shown, e.g. x-axis at 5 mg/ml = 1 mg/ml GMα plus 4 mg/ml GMβ.

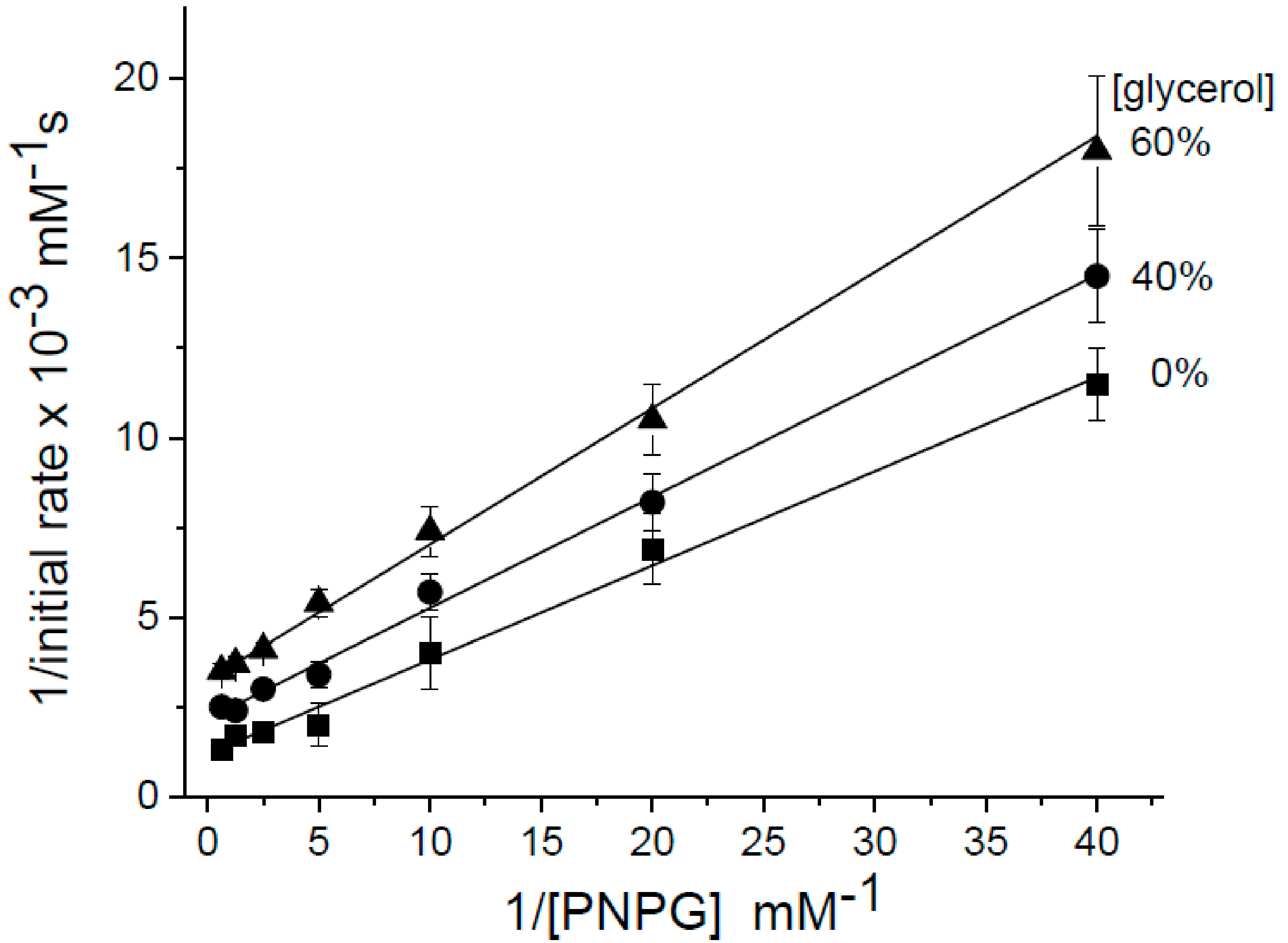

Supplemental Figure S2. Effect of 40% (v:v) and 60% (v:v) glycerol on maltase activity. Solutions are made up using aqueous 20 mM potassium phosphate buffer, pH 7, and data were acquired at 30 ^{o}C. Error bars indicate standard deviations of three different experiments, each run in duplicate.
